# Association between toxic drug events and encephalopathy in British Columbia, Canada: a cross-sectional analysis

**DOI:** 10.1186/s13011-023-00544-z

**Published:** 2023-07-07

**Authors:** Chloé G. Xavier, Margot Kuo, Roshni Desai, Heather Palis, Gemma Regan, Bin Zhao, Jessica Moe, Frank X. Scheuermeyer, Wen Qi Gan, Soha Sabeti, Louise Meilleur, Jane A. Buxton, Amanda K. Slaunwhite

**Affiliations:** 1 BC Centre for Disease Control, 655 West 12th Avenue, Vancouver, BC V5Z 4R4 Canada; 2 Overdose Emergency Response Centre, Ministry of Mental Health and Addictions, 655 West 12th Avenue, Vancouver, BC V5Z 4R4 Canada; 3 Department of Psychiatry, University of British Columbia, 2255 Wesbrook Mall, Vancouver, BC V6T 2A1 Canada; 4 Faculty of Medicine, University of British Columbia, 317—2194 Health Sciences Mall, Vancouver, BC Canada; 5 Woodward Instructional Resource Centre, Vancouver, BC V6T 1Z3 Canada; 6 Department of Emergency Medicine, University of British Columbia, 2775 Laurel Street, Vancouver, BC V5Z 1M9 Canada; 7 Department of Emergency Medicine, Vancouver General Hospital, 899 West 12th Avenue, Vancouver, BC V5Z 1M9 Canada; 8 St Paul’s Hospital and the Department of Emergency Medicine, University of British Columbia, 2775 Laurel Street, Vancouver, BC V5Z 1M9 Canada; 9 Centre for Health Evaluation & Outcomes Sciences, St Paul’s Hospital, 588-1081 Burrard Street, Vancouver, BC V6Z 1Y6 Canada; 10 Health Surveillance, First Nations Health Authority, 100 Park Royal S, West Vancouver, BC V7T 1A2 Canada; 11 School of Population and Public Health, University of British Columbia, 2206 E Mall, Vancouver, BC V6T 1Z3 Canada

**Keywords:** Drug overdose, Opioid, Encephalopathy, Brain Injury, Epidemiology, Addiction Medicine

## Abstract

**Background:**

Encephalopathy can occur from a non-fatal toxic drug event (overdose) which results in a partial or complete loss of oxygen to the brain, or due to long-term substance use issues. It can be categorized as a non-traumatic acquired brain injury or toxic encephalopathy. In the context of the drug toxicity crisis in British Columbia (BC), Canada, measuring the co-occurrence of encephalopathy and drug toxicity is challenging due to lack of standardized screening. We aimed to estimate the prevalence of encephalopathy among people who experienced a toxic drug event and examine the association between toxic drug events and encephalopathy.

**Methods:**

Using a 20% random sample of BC residents from administrative health data, we conducted a cross-sectional analysis. Toxic drug events were identified using the BC Provincial Overdose Cohort definition and encephalopathy was identified using ICD codes from hospitalization, emergency department, and primary care records between January 1st 2015 and December 31st 2019. Unadjusted and adjusted log-binomial regression models were employed to estimate the risk of encephalopathy among people who had a toxic drug event compared to people who did not experience a toxic drug event.

**Results:**

Among people with encephalopathy, 14.6% (n = 54) had one or more drug toxicity events between 2015 and 2019. After adjusting for sex, age, and mental illness, people who experienced drug toxicity were 15.3 times (95% CI = 11.3, 20.7) more likely to have encephalopathy compared to people who did not experience a drug toxicity event. People who were 40 years and older, male, and had a mental illness were at increased risk of encephalopathy.

**Conclusions:**

There is a need for collaboration between community members, health care providers, and key stakeholders to develop a standardized approach to define, screen, and detect neurocognitive injury related to drug toxicity.

**Supplementary Information:**

The online version contains supplementary material available at 10.1186/s13011-023-00544-z.

## Background

A non-fatal toxic drug event (overdose) can result in a partial (hypoxic) or complete loss of oxygen (anoxic) to the brain [1]. Encephalopathy related to a non-fatal toxic drug event, or as a result of long-term substance use issues, can be categorized as a non-traumatic acquired brain injury or toxic encephalopathy [2, 3]. Toxic leukoencephalopathy has been described to occur from various types of licit and illicit substances consumed through inhalation, ingestion, or injection [4]. Severe cases of toxic encephalopathy can develop into toxic leukoencephalopathy [1, 4, 5], delayed post-hypoxic leukoencephalopathy [6–10], and amnesia [11–13]. It has been demonstrated to impact memory, executive functions and psychomotor abilities; ability to concentrate and recall information; speech; the visual system; and, in severe cases, lead to coma or death [1, 6, 7, 11, 12, 14]. Symptoms may not appear until three or more weeks following the event and can last over one year [6, 14], and most people hospitalized following a drug toxicity are discharged within 24 to 48 hours which is often insufficient time for neurocognitive symptoms to appear [15]. This delayed onset of symptoms may suggest underdiagnosing of toxic encephalopathy among people who experienced drug toxicity [6].

Clinical case reports [5, 9, 11, 13, 14], information from community members [3], and media sources [16, 17] have identified encephalopathy as a significant health issue among people who experienced drug toxicity. In April 2016, the Government of British Columbia (BC), Canada declared a public health emergency in response to the rise in toxic drug events [18]. Between January 1st, 2015 and December 31st, 2019 over 30,000 people had a non-fatal toxic drug event [19]. Population level studies that estimate the prevalence of toxic drug events among people who have encephalopathy are limited [20]. Having an estimate of the prevalence of encephalopathy and understanding the co-occurrence of encephalopathy and drug toxicity is critical for understanding the health and long-term care implications of the toxic drug public health emergency. The aim of this analysis was to (1) estimate the prevalence of drug toxicity among people who had encephalopathy, and (2) examine the association between drug toxicity and encephalopathy in BC, Canada, using the BC Provincial Overdose Cohort (BC-ODC).

## Methods

### Study sample and design

The BC-ODC is a linked administrative health dataset which includes health care records, socio-demographic information, and data on all fatal and non-fatal toxic drug events where health care was accessed [19]. A 20% random sample of BC residents registered for universal health care offered through provincial health insurance is included in and linked to the BC-ODC. The 20% random sample and drug toxicity cases in the BC-ODC are linked to provincial ambulatory service, provincial drug poisoning information center, hospitalization and emergency department, physician and outpatient billing, coroners service, vital statistic deaths, chronic disease registry, social assistance payment, and provincial incarceration data from January 1st, 2010 to December 31st, 2019 using de-identified unique personal health numbers. More detailed information on inclusion criteria and the development of the BC-ODC can be found elsewhere [19]. The 20% random sample of BC residents was the study sample for this analysis. A cross-sectional study design was used to examine the association between toxic drug events and encephalopathy.

### Drug toxicity cases

People were classified as drug toxicity cases if they had any record of a fatal or non-fatal toxic drug event between January 1st, 2015 to December 31st, 2019, i.e. all years of available drug toxicity data included in the BC-ODC. Additional information on the BC-ODC case definition can be found in Supplemental Tables 1 and in MacDougall et al. [19].

### Encephalopathy diagnosis

Encephalopathy was identified from records of hospitalization (Discharge Abstract Database [21]), emergency department visits (National Ambulatory Care Reporting System), and outpatient billing (Medical Services Plan [22]) records in the 20% population sample between January 1st, 2015 and December 31st, 2019 using the International Statistical Classification of Diseases (ICD) codes. Using health care records from January 1st, 2010 to December 31st, 2019 we excluded all people who had an encephalopathy ICD code prior to January 1st, 2015 or an encephalopathy diagnostic record before the first recorded toxic drug event in the BC-ODC. Since there are no studies which validate the use of ICD codes to identify toxic encephalopathy [20], we used a definition with high specificity and included: anoxic brain injury, toxic encephalopathy, and encephalopathy unspecified (Supplemental Table 2); the definition from Morrow et al. [20]. To examine diagnostic codes in MSP, we converted the ICD-10 to ICD-9 codes.

### Population characteristics

Demographic, mental health, and overdose characteristics were examined using data included in the BC-ODC. Age and sex were available from the Client Roster database which has up-to-date demographic information on people registered for provincial health insurance in BC. Only people with complete Client Roster records from 2015 to 2019, and with sex and age information were included. Mental illness and substance use disorder (SUD) (excluding alcohol use disorder) were defined as two outpatient records (MSP) within one year, or one hospitalization record (DAD) between January 1st, 2015 and December 31st, 2019 using ICD-9 and ICD-10 codes (Supplemental Table 3), as outlined by the BC Ministry of Health [23]. Mental illness included anxiety, depression, schizophrenia, bipolar disorder, and stress/adjustment disorder.

### Statistical analysis

Chi-square tests of association were used to compare descriptive characteristics. Unadjusted and adjusted log-binomial regression models were used to estimate the risk of encephalopathy among people who had a toxic drug event compared with people who did not. Prevalence ratios (PRs) and 95% confidence intervals (CIs) were calculated in both the unadjusted and adjusted log-binomial model. All statistical tests were conducted in SAS EG 8.3 and at α = 0.05. SUDs were included in the descriptive statistics but were excluded from the unadjusted and adjusted models.

## Results

The prevalence of drug toxicity among people who had encephalopathy was 14.6%. Of the 824,165 people included in the study sample, 369 were identified as having encephalopathy and 5,357 had one or more toxic drug event. Of people who had one or more toxic drug events, 1.0% had an encephalopathy diagnostic record. A higher proportion of people with encephalopathy were 50 years or older (63.4%), male (63.4%), had a mental illness or SUD (61.0%), and had a fatal toxic drug event (2.2%) compared to people who did not have encephalopathy (Table 1).

**Table 1:** Characteristics by encephalopathy diagnostic code (column %)

	Total (N = 824,165)		Encephalopathy diagnosis (N = 369)		No encephalopathy diagnosis (N = 823,796)		
	N	%	N	%	N	%	p-value
Drug toxicity event (overdose), fatal or non-fatal							
Yes	5,357	0.6	54	14.6	5,303	0.6	p < 0.01
No	818,808	99.4	315	85.4	818,493	99.4	
**Age** ^**a**^							
<30 years	197,378	23.9	41	11.1	197,337	24.0	p < 0.01
30–39 years	149,377	18.1	31	8.4	149,346	18.1	
40–49 years	141,428	17.2	63	17.1	141,356	17.2	
≥ 50 years	335,991	40.8	234	63.4	335,757	40.8	
**Sex**							
Female	414,959	50.3	135	36.6	414,824	50.4	p < 0.01
Male	409,206	49.7	234	63.4	408,972	49.6	
**Mental illness** ^**b**^							
No SUD or mental illness	677,154	82.2	144	39.0	677,010	82.2	p < 0.01
SUD	7,145	0.9	22	6.0	7,123	0.9	
Mental illness	128,312	15.6	132	35.8	128,180	15.6	
SUD and mental illness	11,554	1.4	71	19.2	11,483	1.4	
**Number of drug toxicity events**							
1 toxic drug event	3,623	0.4	35	9.5	3,588	0.4	p < 0.01
2 or more toxic drug events	1,734	0.2	19	5.2	1,715	0.2	
None	818,808	99.4	315	85.4	818,493	99.4	
**Fatal drug toxicity**							
Yes	229	0.0	8	2.2	221	0.0	p < 0.01
No	823,939	100.0	361	97.8	823,575	100.0	

^a^ Age January 1st, 2015; ^b^ Diagnosed 2015–2019; SUD: substance use disorder

**Fig. 1:**
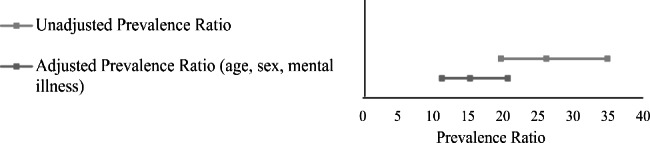
Forest plot of the unadjusted and adjusted association of drug toxicity and encephalopathy

After adjusting for sex, age, and mental illness, people who experienced drug toxicity were 15.3 times (95% CI = 11.3, 20.7) more likely to have encephalopathy compared to people who did not experience drug toxicity (Table 2). In both adjusted and unadjusted models, people who were 40 years and older, male, and had a mental illness were at increased risk of encephalopathy (Table 2 and Fig. 1).

**Table 2:** Association of drug toxicity and encephalopathy

	Unadjusted, PR (95% CI)	Adjusted, PR (95% CI)
Drug toxicity event (overdose)		
No	*Reference*	*Reference*
Yes	26.2 (19.7–34.9)	15.3 (11.3–20.7)
**Age**		
<30 years	*Reference*	*Reference*
30–39 years	1.0 (0.6–1.6)	0.9 (0.6–1.5)
40–49 years	2.1 (1.4–3.2)	2.1 (1.4–3.2)
≥ 50 years	3.4 (2.4–4.7)	3.9 (2.8–5.5)
**Sex**		
Female	*Reference*	*Reference*
Male	1.8 (1.4–2.2)	2.0 (1.6–2.5)
**Mental illness** ^**a**^		
No mental illness diagnosis	*Reference*	*Reference*
Mental illness diagnosis	6.0 (4.9–7.3)	5.4 (4.4–6.8)

^a^Mental illness does not include substance use disorder (SUD)

PR: Prevalence ratio; CI: confidence interval

## Discussion

This study aimed to conduct a preliminary analysis of the risk of encephalopathy among people who had a toxic drug event in a population-based sample. The prevalence of drug toxicity among people with encephalopathy between January 1st, 2015 and December 31st, 2019 was 15%. After adjusting for demographic factors and mental illness, we demonstrated that people who experienced a toxic drug event were 15 times more likely to have encephalopathy than people who did not experience drug toxicity. These findings highlight the potential long-term health outcomes that persons may experience following drug toxicity, along with the need to develop health care services and outreach programs specifically for people who experience toxic drug events and a neurocognitive injury.

Most studies which examine toxic encephalopathy among people who experienced drug toxicity are clinical case reports [4, 5, 11, 13, 14, 24–28] suggesting that encephalopathy is an important health concern among this population. Though population level studies that estimate drug toxicity-related encephalopathy are limited [20], and no existing literature attempts to estimate the co-occurrence of toxic encephalopathy and drug toxicity, there are some consistencies between our findings and the available literature. A study by Morrow et al. reported that 3% of patients admitted to hospitals from 2006 to 2015 in BC, Canada for accidental opioid overdose had a record of encephalopathy as a result of an overdose event [20]. In the study, toxic drug events are only identified using hospitalization, inpatient, and outpatient records therefore underestimates the number of people who had atoxic drug event. Older age has also been described as an important factor that affects the severity of recovery from encephalopathy [15, 25]. Consistent with the available literature, our study found a high proportion of people with encephalopathy died from drug toxicity within the study timeframe [1, 25].

Though it is not possible to ascertain temporality in the association between toxic drug events and encephalopathy given the cross-sectional nature of our analysis, most of the available literature outlines directionality where drug toxicity leads to encephalopathy. Increasingly, the literature is highlighting the potential health implications of non-fatal toxic drug events on neuropathology and cognitive health outcomes [29, 30]. Diagnosis of encephalopathy is complex as there may be delayed onset of symptoms [6, 14], clinical presentations vary, and there rarely exists a measure of pre-existing neurological function [5, 15]. The lack of standardized mechanism to identify drug toxicity-related encephalopathy using clinical or radiological methods [1] suggest that our study may underestimate the prevalence of encephalopathy. There is a need for collaboration between people who use drugs, community-based organizations (such as harm reduction sites and shelters), health care providers, and other key stakeholders to develop a standardized approach to define, screen, and detect neurocognitive injury among people experiencing drug toxicity.

Encephalopathy, particularly if undiagnosed or untreated, can severely affect cognitive capabilities [1, 25], increase the likelihood of subsequent toxic drug events, and impact social activities and livelihoods. And, may compound existing barriers, such as access to housing, employment, community supports, and increasing experiences of stigma [3]. Several studies have outlined significant changes in a person’s level of independence following encephalopathy from drug toxicity [15, 25], which has important implications for health and long-term care provision. Studies have also described the positive impacts of rehabilitation among people with drug toxicity-related encephalopathy, such as some recovery of memory and executive function as well as ability to live independently [5, 7, 8, 25], highlighting the importance of screening and access to rehabilitation services for this population. While there is limited research and evaluation on programs supporting people with encephalopathy following a toxic drug event, emerging services suggest that using a harm reduction, person-centered approach is critical. In BC, the Vancouver Coastal Health Authority has developed a Cognitive Assessment and Rehabilitation for Substance Use program at Richmond Hospital that aims to address this gap.

Community members, including harm reduction and front-line workers, are seeing the impacts of the drug toxicity crisis on neurocognitive health yet are often not included in service planning for people experiencing toxic drug events. Developing programs and services for people experiencing encephalopathy should center the expertise of people who use drugs and community members particularly since an increasing number of toxic drug events are reversed in community without interaction with health care. Front-line workers, community members, and other first responders should also be educated on the symptoms of and included in the development of screening practices for encephalopathy among people who had a toxic drug event. There is also a need for qualitative research to understand the experiences of people with encephalopathy and among front-line workers supporting this population and longitudinal analyses estimating the incidence of encephalopathy among people who experienced drug toxicity.

### Limitations

Not every person who experiences drug toxicity accesses health care and many toxic drug events are reverse in community, therefore not all toxic drug events, or cases of encephalopathy are reported in these data. Due to the cross-sectional nature of the analysis, we are not able to determine the temporal relationship between encephalopathy and drug toxicity. Though there seems to be a strong association, it is unclear whether people who experienced drug toxicity are at increased risk of encephalopathy or if people with encephalopathy are at increased risk of a toxic drug event, or both. Data on intervention type (e.g. naloxone administration, oxygen) and time between drug toxicity event and intervention were not available across all data sources therefore it could not be included in this analysis. Measuring encephalopathy using administrative data can be challenging as diagnostic codes likely capture the most severe cases and underestimate the burden of mild to moderate cases, particularly given the lack of screening. The use of ICD codes to examine encephalopathy likely result in low specificity and impacts the ability to identify encephalopathy among the study population. Our findings of a high proportion of drug toxicity deaths among people with an encephalopathy diagnostic code could be attributable to an over-representation of severe encephalopathy cases but may also reflect riskier drug using behaviours among people who have encephalopathy.

## Conclusions

Future research should develop a standard definition for drug toxicity-related encephalopathy and validate the use of ICD codes in administrative data for measuring encephalopathy. The findings highlight that the toxic drug (overdose) crisis and the toxic drug supply not only contribute to the large and preventable loss of life in BC, Canada but may also result in severe hypoxic and anoxic events for persons who use drugs. An urgent and collaborative effort is needed across health and social services to advance screening and diagnosis of encephalopathy and enhance support services for people who experienced toxic drug events.

## Electronic supplementary material

Below is the link to the electronic supplementary material.

Supplementary Material 1

## Data Availability

The data used in this study is not publicly available due to privacy considerations. However, researchers can request access to the BC Provincial Overdose Cohort via annual calls for proposals through Population Data BC.

## List of abbreviations

BC: British Columbia
BC-ODC: BC Provincial Overdose Cohort
DAD: Discharge Abstract Database
ICD: International Statistical Classification of Diseases
MSP: Medical Services Plan
SUD: Substance use disorder
