# Supplementary material for: Association between toxic drug events and encephalopathy in British Columbia, Canada: a cross-sectional analysis
Source: Subst Abuse Treat Prev Policy. 2023 Jul 7;18:42. doi: 10.1186/s13011-023-00544-z (PMC10329314; doi:10.1186/s13011-023-00544-z)
Supplement: Supplementary file 1 — Supplementary Material 1 [file 13011_2023_544_MOESM1_ESM.docx]

| **Supplemental Table 1.** ICD-10 and ICD-9 Codes for Identification of Drug Poisoning Cases, only significant diagnosis codes   \|  \| **Hospitalizations, emergency department visits, Vital Statistics**  **(ICD-10)** \| **Primary care (ICD-9)** \|  \| \| --- \| --- \| --- \| --- \| \| Poisoning by, adverse effect of and underdosing of opium Poisoning by and adverse effect of heroin  Poisoning by, adverse effect of and underdosing of other opioids  Poisoning by, adverse effect of and underdosing of methadone Poisoning by, adverse effect of and underdosing of other synthetic narcotics  Poisoning by, adverse effect of and underdosing of other and unspecified narcotics \| T40.0  T40.1  T40.2  T40.3  T40.4  T40.6 \|  \|  \| \| Poisoning by opiates and related narcotics  Poisoning by heroin  Poisoning by methadone  Poisoning by other opiates and related narcotics  Accidental poisoning by heroin \|  \| 965.00  965.01  965.02  965.09  E850.00 \|  \|   **Supplemental Table 2.** ICD codes for identification of encephalopathy cases, any documented diagnosis codes | | |
| --- | --- | --- | --- | --- | --- | --- | --- | --- | --- | --- | --- | --- | --- | --- |
|  | **Hospitalizations, emergency department visits (ICD-10)** | **Primary care (ICD-9)** |
| Encephalopathy, including anoxic brain damage  Toxic encephalopathy  Unspecified encephalopathy | G93.1  G92  G93.4 |  |
| Encephalopathy, including anoxic brain damage  Toxic encephalopathy  Toxic encephalitis and encephalomyelitis  Toxic myelitis  Unspecified encephalopathy |  | 348.1  323.71  323.72  349.82  348.30 |

**Supplemental Table 3.** ICD codes for mental illness and substance use disorder diagnoses, any documented diagnosis codes

|  | **Hospitalizations**  **(ICD-10)** | **Primary care (ICD-9)** |
| --- | --- | --- |
| **Anxiety disorder**  Phobia anxiety disorders  Other anxiety disorders  Neurotic disorders | F40  F41 | 300^b^ |
| **Depression**  Major depressive disorder, single episode  Major depressive disorder, recurrent  Dysthymic disorder  Neurotic depression  Depressive disorder, not elsewhere classified | F32  F33  F34.1 | 300.4  311 |
| **Schizophrenia**  Schizophrenia  Schizotypal disorder  Delusional disorders  Brief psychotic disorders  Shared psychotic disorder  Schizoaffective disorders  Other psychotic disorder  Unspecified psychosis  Schizophrenic psychoses  Paranoid states  Other nonorganic psychoses | F20  F21  F22  F23  F24  F25  F28  F29 | 295  297  298 |
| **Bipolar disorder**  Manic episode  Bipolar disorder  Persistent mood/affective disorders  Unspecified mood/affective disorder  Affective psychoses | F30  F31  F34^c^  F39 | 296 |
| **Stress and adjustment disorders**  Reaction to severe stress, and adjustment disorders  Acute reaction to stress  Adjustment reaction | F43 | 308  309 |

^a^ does not include 305.0; ^b^ does not include 300.4; ^c^ does not include F34.1
